# Molluscicidal efficacies of different formulations of niclosamide: result of meta-analysis of Chinese literature

**DOI:** 10.1186/1756-3305-3-84

**Published:** 2010-09-07

**Authors:** Guo-Jing Yang, Wei Li, Le-Ping Sun, Feng Wu, Kun Yang, Yi-Xin Huang, Xiao-Nong Zhou

**Affiliations:** 1 Jiangsu Institute of Parasitic Diseases, Meiyuan Yangxiang 117, Wuxi 214064, Jiangsu, People's Republic of China; 2 National Institute of Parasitic Diseases, Chinese Center for Disease Control and Prevention, 207 Rui Jin Er Road, Shanghai 200025, People's Republic of China

## Abstract

The control efforts on *Oncomelania hupensis*, the intermediate snail host of *Schistosoma japonicum*, cannot be easily excluded from the integrated approach of schistosomiasis control in China. Application of chemical compounds, molluscicides, in snail habitats is a common method for snail control in addition to environmental modification. We conducted a systematic review and meta-analysis to assess the molluscicidal effects of the currently recommended 50% niclosamide ethanolamine salt wettable powder and a new 4% niclosamide ethanolamine salt powder developed by Chinese researchers. Literature was searched from three Chinese databases, i.e. Chinese Biomedical Database, VIP Database and Wanfang Database, on field mollusciciding trials of niclosamide in China (from January 1, 1990 to April 1, 2010). Molluscicidal effects on reduction of snail population of the 50% or 4% niclosamide formulations in field trial were evaluated 3 days, 7 days or 15 days post-application. Out of 90 publications, 20 papers were eventually selected for analysis. Publication bias and heterogeneity tests indicated that no publication bias existed but heterogeneity between studies was present. Meta-analysis in a random effect model showed that the snail mortality of 3, 7 and 15 days after spraying the 50% niclosamide ethanolamine salt wettable powder were 77% [95%CI: 0.68-0.86], 83% [95%CI: 0.77-0.89], and 88% [95%CI: 0.82-0.92], respectively. For the 4% niclosamide ethanolamine salt powder, the snail mortality after 3, 7 and 15 days were 81% [95%CI: 0.65-0.93], 90% [95%CI: 0.83-0.95] and 94% [95%CI: 0.91-0.97], respectively. Both are good enough to be used as molluscicides integrated with a schistosomiasis control programme. The 4% niclosamide ethanolamine salt powder can be applied in the field without water supply as the surrogate of the current widely used 50% niclosamide ethanolamine salt wettable powder. However, to consolidate the schistosomiasis control achievement gained, it is necessary to continuously perform mollusciciding more than twice annually in the field.

## Introduction

The transmission records of schistosomiasis japonica, a disease prevalent in the Yangtze River valley and southern part of China, can be traced back over 2,000 years [1]. The distribution of the disease was determined by the distribution of its exclusive intermediate host snail, i.e. *Oncomelania hupensis *[2-4]. Geographically, *O. hupensis *habitats can be divided into three ecological types: (i) lake and marshland regions; (ii) hilly and mountainous regions; and (iii) plain regions with water networks [2]. Large-scale disease control programmes were initially carried out in the 1950s by the Chinese government from central to local levels, putting a strong emphasis on snail control, including environmental modification and the application of chemical molluscicides [1,5]. The achievements were consolidated through a 10-year World Bank Loan Project (WBLP) for schistosomiasis control starting in 1992. Prevalence and morbidity reached the lowest level in 2000, which is partially explained by mass chemotherapy [3,4,6]. Soon after the ending of WBLP in 2001, the disease emerged or re-emerged in some regions [7], which drew attention of the government again and a new integrated control program was launched in 2004 with emphasis on health education, access to clean water and adequate sanitation, mechanization of agriculture and fencing of water buffaloes, along with chemotherapy [8,9]. The importance of the mollusciciding in the integrated control of human schistosomiasis has gone through cyclical changes over the past 6 decades. For a time, it was hoped that chemotherapy alone would achieve significant morbidity control. However, many recent studies have shown that control programmes based on chemotherapy alone can provide only a temporary reduction in transmission [10,11]. Snail control, therefore, is important in integrated schistosomiasis control as the disease is environment-related [12,13]. Favourable environmental conditions required for disease transmission still exist in endemic areas, and it would be difficult to maintain the current low level of infection if dense populations of the intermediate host persist [14]. In addition, ecological transformations bring about new challenges for control, most notably the Three Gorges dam project, and the South-to-North Water Transfer Project as well as global warming [15,16].

Application of chemical compounds in snail habitats is a common method for elimination of intermediate host snails, in addition to environmental modification, such as cementing canals, re-adjusting irrigation systems, etc [14,17]. Niclosamide has been recommended by WHO as a sole molluscicide since the 1960s [18] and is still the molluscicide of choice [19]. In China, other synthetic molluscicides, such as sodium pentachlerophenate (NaPCP), were widely used before WBLP. Due to the safety concerns and severe problems of environmental pollution, NaPCP has been banned from use in China [20]. Niclosamide is the only molluscicide applied currently in the field for snail control in China, although there have been many formulations of niclosamide developed over the past few decades. One niclosamide formulation is a 50% niclosamide paste known as schistosomiasis-67 paste, and has contributed to snail control at low cost. But this formula as a paste form easily clogs and in turn blocks the nozzle of the sprayer while applying in the field. Hence, special methods of transportation and packaging were required which made the field application inconvenient. Chen et al. [20] later prepared a new formulation of niclosamide named niclosamide salt wettable powder with a water suspension rate of 60%. The molluscicidal effect of this formulation is twice as high as that of the 50% paste. Later on, the amino ethanol salt of niclosamide, called Bayluscide, slightly improved the water solubility of niclosamide. In 1986, the Institute of Parasitic Diseases, Sichuan Academy of Medical Sciences successfully developed a novel long-lasting molluscicide, i.e. a 10% controlled-release niclosamide formulation. In 1992, the 50% niclosamide ethanolamine salt wettable powder was introduced and produced in China during WBLP due to its excellent suspension in water and strong molluscicidal effect. It is the most commonly used molluscicide in China at present. However, water is required for application of the formulation limiting its use in regions lacking water. Recently, a 4% niclosamide ethanolamine salt powder was developed as a supplement of niclosamide ethanolamine salt [21]. It can overcome the difficulty of limited water resources, and will further expand the application range of niclosamide.

Many similar studies have been carried out to evaluate the molluscicidal effect of different molluscicides in field trials in China. Due to the variation of environmental settings, a large variety of the molluscicidal effects on snail mortality (40-100%) no doubt impact the government decision makers to formulate the snail control strategy. Therefore, we conducted a systematic review and meta-analysis to assess the molluscicidal effect of the currently recommended 50% niclosamide ethanolamine salt wettable powder and of the new 4% niclosamide ethanolamine salt powder.

## Methods

A systematic search of the Chinese literature, including documents published from January 1, 1990 to April 1, 2010, was conducted to capture data on molluscicidal effect on snail mortality by niclosamide in field trials in China.

### Search strategy and data source

Three major Chinese literature databases, namely Chinese Biomedical Database, VIP Database and Wanfang Database, were jointly searched for data pertaining to molluscicidal effect of the 50% niclosamide ethanolamine salt wet powder or the 4% niclosamide ethanolamine salt powder in field trials in China. Since the databases are domestic, the language of publication was Chinese. We used the terms "niclosamide", or "Luomiecide (the 50% niclosamide ethanolamine salt wettable powder)", or "Qiangluocide (the 4% Niclosamide ethanolamine salt powder)" and "molluscicide", or "snail control". The abstracts of each screened publication were read carefully.

### Criteria of inclusion and exclusion

Both inclusion criteria and exclusion criteria for searching published literature were settled in the first stage of the meta-analysis. The inclusion criteria of published literature were as follows: (1) the 50% niclosamide ethanolamine salt wettable powder or the 4% niclosamide ethanolamine salt powder was used; (2) molluscicidal experiments carried out in marshland; (3) the used dose of niclosamide in the field was 2 g/m^2^; (4) molluscicidal effects on mortality of *O. hupensis *were evaluated 3 days, 7 days or 15 days post drug application.

The exclusion criteria of published literature were as follows: (1) application dosage of 2 g/m^2 ^was not standardized or used in a wrong approach; (2) no original data, such as number of observations and number of dead snails, were published in the paper; (3) review article, or books or conference abstracts; (4) editorials or letters to the editors without original data.

### Analysis of publication bias

The conclusion we draw may be distracted if we do not consider the bias that may exist from the literature review. Among all the biases, the publication bias is more difficult to control and adjust for. Identification of the bias is particularly important for the future adjustment. We applied "Metabias" function to test for funnel plot asymmetry based on a linear regression method, where the method "linreg" is the statistical test based on a weighted linear regression of the treatment effect on its standard error [22].

### Heterogeneity test

Heterogeneity in meta-analysis refers to the variation in study outcomes between studies. The classical measure of heterogeneity is Cochran's Q. The I² statistic describes the percentage of variation across study that is due to heterogeneity rather than chance [23,24], with its estimated formula: I² = 100% × (Q-df)/Q. If there is very little variation between studies, I² will be low and a fixed effects model might be appropriate. An alternative approach, 'random effects', allows the study outcomes to vary in a normal distribution between studies. Many investigators consider the random effects approach to be a more natural choice than the fixed effects.

All statistical analyses were done in *R *Package [25]. All ratios were double arcsine transformed by Freeman-Tukey method. Forest plot, also called confidence interval plot, is drawn by the function of "Forest".

## Results

### Literature searched

**Figure 1:**
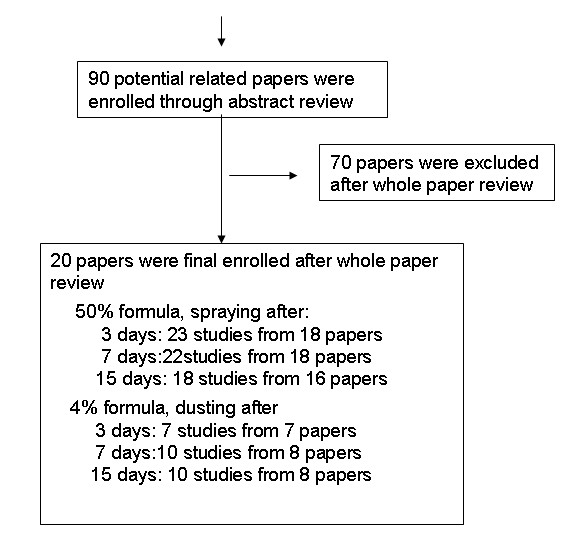
**The flow diagram of paper review process**.

A total of 90 documents, with potential original data on molluscicidal effect by niclosamide in field trials in China, were identified. The flow diagram in Figure 1 shows the review process, including the number of papers identified and number for exclusion of documents according to criteria. A total of 23 [21,26-42], 22 [21,26-32,34-43] and 18 [21,26-32,34,35,37,38,40-42] studies from 18, 18 and 15 papers pertaining to molluscicidal effect of the 50% formulate 3, 7 and 15 days after applying in the field were included, respectively. For the 4% one, 7 [21,26-30], 10 [21,26-30,44,45] and 10 [21,26-30,44,45] studies from 7, 8 and 8 papers were enrolled, respectively.

### Publication bias

**Figure 2:**
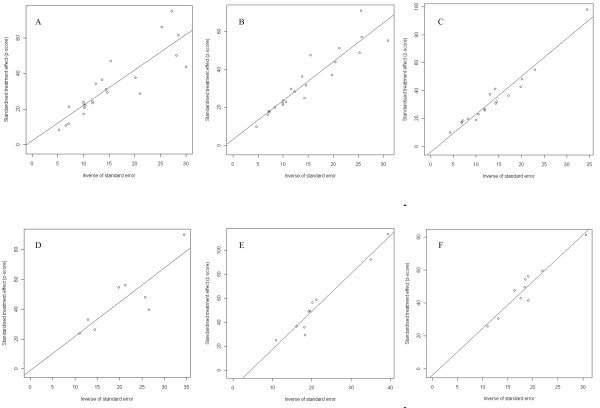
**Linear regression test of funnel plot asymmetry for the 50% molluscicides niclosamide ethanolamine salt wettable powder 3 days (A), 7 days (B) and 15 days (C) post spraying, and for the 4% niclosamide ethanolamine salt powder 3 days (D), 7 days (E) and 15 days (F) post dusting, respectively**. (A: *t *= 0.5716, df = 21, *p*-value = 0.5737; B: *t *= 1.05, df = 20, *p*-value = 0.30; C: *t *= -1.66, df = 16, *p*-value = 0.12; D: *t *= -0.27, df = 5, *p*-value = 0.80; E: *t* = -2.25, df = 8, *p*-value = 0.06; F: *t *= -0.62, df = 8, *p*-value = 0.56)

According to the metabias analysis results, all publications regarding two molluscicides indicated no publication bias with all *p *value higher than 0.05. The linear regression figures showed all selected publications normally scattered around the regression line (Figure 2).

### Heterogeneity test

The heterogeneity test indicated the existence of heterogeneity between studies. The I^2 ^value of the 50% and 4% formulations studies 3, 7 and 15 days post application are all higher than 95% (Table 1).

**Table 1:** Heterogeneous test results of the 50% niclosamide ethanolamine salt wettable powder and the 4% niclosamide ethanolamine salt powder (Freeman-Tukey double arcsine transformation used for proportions)

Post-spraying (day)	tau^2^	H	I^2^	Q	df	*p-value*
50% formulation						
3	0.27	8.66 [7.97; 9.42]	98.70% [98.40%; 98.90%]	1651.76	22	**< 0.0001**
7	0.14	6.12 [5. 50; 6.81]	97.30% [96.70%; 97.80%]	785.58	21	**< 0.0001**
15	0.10	4.91 [4.29; 5.63]	95.90% [94.60%; 96.80%]	410.13	17	**< 0.0001**
4% formulation						
3	0.29	11.97 [10.54; 13.6]	99.30% [99.10%; 99.50%]	859.97	6	**< 0.0001**
7	0.14	8.59 [7. 54; 9.79]	98.60% [98.20%; 99.00%]	664.08	9	**< 0.0001**
15	0.06	4.80 [3.97; 5.80]	95.70% [93.70%; 97.00%]	207.36	9	**< 0.0001**

### Meta analysis

**Figure 3:**
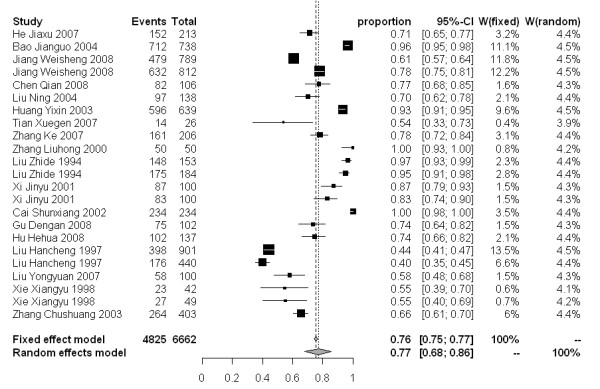
**Molluscicidal effects (individual and pooled results) of the 50% niclosamide ethanolamine salt wettable powder 3 days after spraying**.

**Figure 4:**
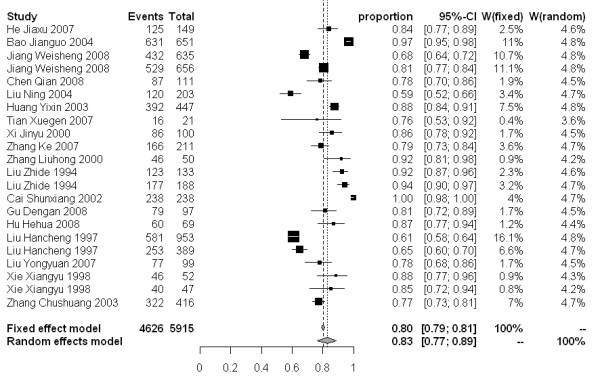
**Molluscicidal effects (individual and pooled results) of the 50% niclosamide ethanolamine salt wettable powder 7 days after spraying**.

**Figure 5:**
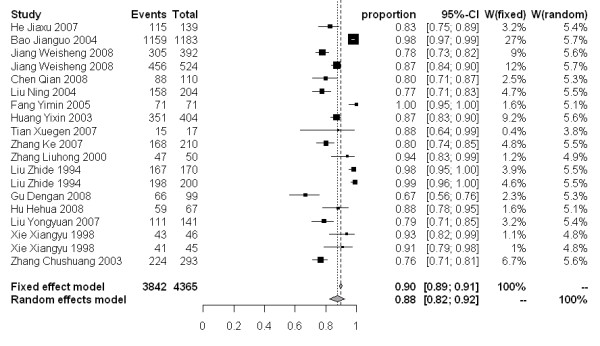
**Molluscicidal effects (individual and pooled results) of the 50% niclosamide ethanolamine salt wettable powder 15 days after spraying**.

**Figure 6:**
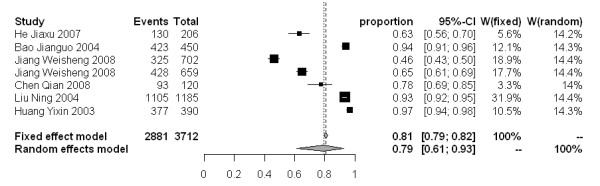
**Molluscicidal effects (individual and pooled results) of the 4% niclosamide ethanolamine salt powder 3 days after dusting**.

**Figure 7:**
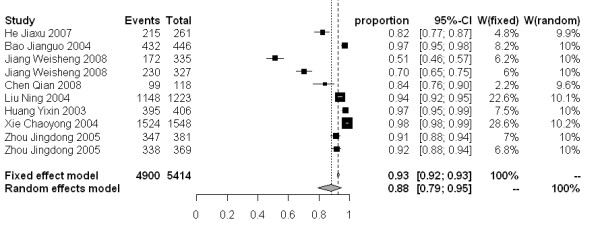
**Molluscicidal effects (individual and pooled results) of the 4% niclosamide ethanolamine salt powder 7 days after dusting**.

**Figure 8:**
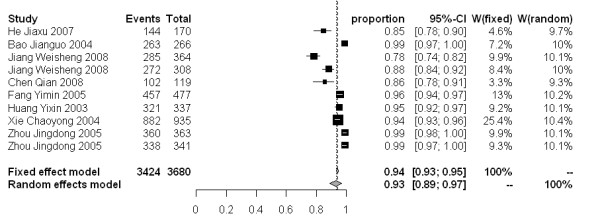
**Molluscicidal effects (individual and pooled results) of the 4% niclosamide ethanolamine salt powder 15 days after dusting**.

The molluscicidal effect on snail mortality of the 50% and 4% formulations presented a wide range of values. For the 50% formulation, snail mortality 3, 7 and 15 days after spraying ranged from 40-100%, 59-100% and 67-100%, respectively. While for the 4% formulation, the snail mortality 3, 7 and 15 days after dusting spanned from 46-97%, 51-98% and 78-99%, respectively. The snail mortality of the 50% formulation after 3, 7 and 15 days in the random effect model were 77% [95% CI: 0.68-0.86], 83% [95% CI: 0.77-0.89], and 88% [95% CI: 0.82-0.92], respectively. For the 4% formulation, the snail mortality 3, 7 and 15 days post application were 79% [95% CI: 0.61-0.93], 88% [95% CI: 0.79-0.95] and 93% [95% CI: 0.89-0.97], respectively (Figure 3-8).

## Discussion

The present study, to our knowledge, represents the first systematic review and meta-analysis on molluscicidal effect of different formulations of molluscicides, i.e. the 50% niclosamide ethanolamine salt wettable powder, and the 4% niclosamide ethanolamine salt powder, applied in the field of China. In the present meta-analysis, the publication biases were not detected in all study groups, which strengthens the likelihood that our meta-analysis was successful in including studies of a certain coverage and quality, which may have made results more comparable and reliable. The heterogeneity remained in each study group suggested that the application of the random effect model was optimal for our meta-analysis. Among the two formulations, the 50% niclosamide ethanolamine salt wettable powder has been applied in the field for more than 20 years, and it was quite acceptable for the local use in mollusciciding owing to its excellent suspension in water and strong molluscicidal effect. So far, no evidence of drug resistance has been reported, which indicated the stability of the niclosamide product. But, several reports were on the disadvantages in the application of the 50% niclosamide ethanolamine salt wettable powder, particularly in regions without water supply [29,44]. The 4% niclosamide ethanolamine salt powder is a newly formulated product, and is able to overcome the problems of water shortage as well as of blocking equipment during application.

Our systematic review and meta-analysis identified that the molluscicidal effect of both formulations are suitable for the mollusciciding in schistosomiasis control programme, causing snail mortality over 88% 15 days after application. The molluscicidal effects of the 4% formulation are slightly higher than those of the 50% formulation 3, 7 and 15 days after spraying, respectively. However, the numbers of studies utilized in our meta-analysis for the 50% formulation was almost twice the number of the 4% formulation, which indicated the results of the 50% formulation are more stable and reliable. Therefore, in the future, it is recommended that more field observations on molluscicidal effect of the 4% formulation need to be carried out in various environmental settings.

Currently, schistosomiasis japonica is mainly prevalent in lake and marshland regions and part of the hilly and mountainous regions of China[2]. In the lake and marshlands regions, snails spread out in vast areas of the Yangtze River basin, which are flooded for about 2 to 5 months per year. In the hilly and mountainous regions, the snails are distributed along ditches, irrigation channels and river systems, but are isolated from one another [2,46]. In spite of the 50% formulation as the most frequent used molluscicide over decades in China [20,47], its molluscicidal effect could be impeded due to the shortage of water. While the 4% formulation can become a surrogate of the 50% formulation, with its advantage of application in the areas shortage of water [47]. Therefore, it is convenient for the 50% formulation applied in the lake and marshland region as well as the plain region with water networks owing to the rich water supply, while the application of the 4% formulation in the hilly and mountainous region, where there is difficulty in water access, is more practical and efficient. Unfortunately, in this study, we do not have enough references of the mollusciciding applied in hilly and mountainous region to test the above hypothesis.

Although this systematic review and meta-analysis identified that the molluscicidal effect of both formulations of niclosamide are good enough for the mollusciciding in a schistosomiasis control programme, both formulations cannot produce 100% snail mortality. Meta-analysis results showed that the snail mortality of the 50% and 4% formulations are 88% and 93%, respectively, 15 days after applying. It indicates the low density of *O. hupensis *snails would survive following the one spray in the snail control activity. According to the law of biological growth and development, density dependency (negative feedback) plays an important role for the population dynamics. There is a general consensus among ecologists that small arthropods or invertebrate animals will follow the Gompertz logistic (GL) reproduction model, which means high turn-over rates occurred at low density [48,49]. Therefore, an assumption can be drawn that if mollusciciding is only attempted once by spraying niclosamide in the field, the survival of few snails will re-bounce following GL negative feedback in the coming years, which make mollusciciding as one of control strategies display low cost-effectiveness. In order to consolidate snail control achievement in terms of keeping snail density at a certain lower level or even elimination of *O. hupensis *snails in the field, it is recommended to perform mollusciciding more than twice annually. While studies on the optimal times of annual mollusciciding to eliminate *O. hupensis *over certain years is still unknown and we need further investigations on the ecology of *O. hupensis *to understand its dynamics.

## Competing interests

The authors declare that they have no competing interests.

## Authors' contributions

GJY and XNZ conceived and wrote the first version of the manuscript. GJY, WL, LPS, FW, KY and YXH revised the manuscript. XNZ and GJY finalized the manuscript. All of authors read and approved the final version of the manuscript.
